# The Signaling Molecule Indole Inhibits Induction of the AR2 Acid Resistance System in *Escherichia coli*

**DOI:** 10.3389/fmicb.2020.00474

**Published:** 2020-04-15

**Authors:** Nathaniel Boon, Manpreet Kaur, Amina Aziz, Morissa Bradnick, Kenta Shibayama, Yoko Eguchi, Peter A. Lund

**Affiliations:** ^1^School of Biosciences and Institute of Microbiology and Infection, University of Birmingham, Birmingham, United Kingdom; ^2^Department of Science and Technology on Food Safety, Faculty of Biology-Oriented Science and Technology, Kindai University, Wakayama, Japan

**Keywords:** acid, two component system, indole, *Escherichia coli*, EvgS

## Abstract

Induction of the AR2 acid response system of *Escherichia coli* occurs at a moderately low pH (pH 5.5) and leads to high levels of resistance to pH levels below 2.5 in the presence of glutamate. Induction is mediated in part by the EvgAS two component system. Here, we show that the bacterial signaling molecule indole inhibits the induction of key promoters in the AR2 system and blocks the development of glutamate-dependent acid resistance. The addition of tryptophan, the precursor for indole biosynthesis, had the same effects, and this block was relieved in a *tnaA* mutant, which is unable to synthesize indole. Expression of a constitutively active EvgS protein was able to relieve the inhibition caused by indole, consistent with EvgS being inhibited directly or indirectly by indole. Indole had no effect on autophosphorylation of the isolated cytoplasmic domain of EvgS. This is consistent with a model where indole directly or indirectly affects the ability of EvgS to detect its inducing signal or to transduce this information across the cytoplasmic membrane. The inhibitory activity of indole on the AR2 system is not related to its ability to act as an ionophore, and, conversely, the ionophore CCCP had no effect on acid-induced AR2 promoter activity, showing that the proton motive force is unlikely to be a signal for induction of the AR2 system.

## Introduction

The enteric bacterium *Escherichia coli* has multiple systems that can protect it against low pH, the best characterized of which is the AR2 or GAD system (for recent reviews, see Foster, 2004; Slonczewski et al., 2009; Kanjee and Houry, 2013; Lund et al., 2014; de Biase and Lund, 2015). Genes in this regulon protect *E. coli* against low pH through a range of mechanisms, including reductive decarboxylation of imported glutamate or glutamine and antiport of the product, and the production of periplasmic chaperones that are able to protect proteins from denaturation by low pH. The regulon is subject to a range of complex interacting control mechanisms, the nature of which depend on whether cells are in exponential phase or stationary phase. The actual physical signal that mediates induction of the AR2 system is unknown, but in exponential phase, a particularly important role is played by the two component system EvgAS (Ma et al., 2004; Itou et al., 2009; Burton et al., 2010; Eguchi et al., 2011).

The EvgS sensor kinase is one of five so-called unorthodox sensor kinases in *E. coli*. Upon activation, the EvgS kinase auto-phosphorylates, and the phosphate is transferred to the response regulator EvgA via an internal phosphotransfer relay, a process that has been proposed to increase the sensitivity of sensor kinases as well as reducing their susceptibility to noise (Kim and Cho, 2006; Csikász-Nagy et al., 2011). Phosphorylated EvgA activates expression of YdeO which, in turn, activates expression of GadE. Both GadE and YdeO activate other components of the AR2 pathway, with GadE responsible for activating expression of the decarboxylase proteins GadA and GadB, the glutamine/GABA antiporter GadC, and the periplasmic chaperones HdeA and HdeB. The dynamics of this process are complex but the net effect is the induction of the expression of a large number of genes that act together to enable *E. coli* survival at low pH (typically pH 2.5), a phenomenon often referred to as extreme acid resistance. The usual way in which the EvgAS system is activated experimentally is by short term (typically 30–60 min) incubation of the cells at a moderately low pH of 5.5. The pH range for effective activation is narrow, consistent with the ultra-sensitivity endowed by the internal phosphorelay (Burton et al., 2010; Eguchi et al., 2011; Eguchi and Utsumi, 2014). Whether EvgS is directly detecting the decreased pH or some other property of the cell itself that is changed at low pH is unknown. There is considerable variation in the ability of EvgS from different isolates of *E. coli* to respond to low pH, suggesting the existence of other input signals (Roggiani et al., 2017). Several mutations in *evgS* have been isolated that encode constitutively active forms of EvgS. These turn on genes of the AR2 pathway even at neutral pH. The relevant substitutions are all in the cytoplasmic PAS domain, consistent with a model where this domain mediates interactions in an EvgS dimer that switch it between active and inactive states (Itou et al., 2009; Johnson et al., 2014).

A striking feature of the EvgS sensor kinase is that it has a large periplasmic domain of approximately 61 kDa (out of a total molecular mass of 135 kDa). Structural predictions for this domain reveal that it contains two Venus fly-trap domains, a feature that it shares with several other sensor kinases in pathogenic bacteria, including the well-studied BvgS from *Bordetella pertussis* (Herrou et al., 2010; Dupré et al., 2015a; Sen et al., 2017). Venus fly-trap domains typically show a structural transition from an open to closed conformation when they bind their ligand, a process that in turn can trigger a signal transduction cascade (Mao et al., 1982; Tam and Saier, 1993). It is reasonable to propose that the activity of EvgS and related sensor kinases with large periplasmic domains are modulated by the binding of a periplasmic ligand or ligands, but the nature of these is not known. We therefore screened a range of small molecules (cadaverine, gamma-aminobutyric acid, glycine betaine, indole, ornithine, sarcosine, and spermine) which we considered to be potential candidates for modulators of EvgS activity, based both on consideration of the predicted EvgS periplasmic domain structure (Sen et al., 2017) and knowledge of metabolites produced by *E. coli*. We used a simple promoter probe to assay the effects of these molecules on induction of AR2 promoters at pH 5.5. We show here that the bacterial signaling molecule indole is a potent inhibitor of the exponential phase AR2 acid stress response in *E. coli*, and we provide evidence consistent with this inhibition being a consequence of the inhibition of EvgS activity.

## Materials and Methods

### Strains and Plasmids

The bacterial strains and plasmids used in this study are listed in Supplementary Table S1.

### Growth and Induction Conditions

Cultures were grown at 37°C with shaking at 180 rpm, unless stated otherwise. Growth was monitored by measuring turbidity at 600 nm. All strains were grown in lysogeny broth (LB; 1% w/v tryptone, 0.5% w/v yeast extract, 1% w/v NaCl; pH 7). All plating was done on LB agar (LB + 1.5% w/v agar; pH 7). Inductions were done using M9suppK medium, which is M9 minimal media supplemented with 0.2% casamino acids and either 0.2% glucose or 0.2% glycerol, plus 100 mM KCl (to optimize induction; Eguchi and Utsumi, 2014) and 50 mM MOPS and MES to buffer against pH change. The final pH was adjusted to either 7 or 5.5 with 100 mM HCl. For induction experiments, a single colony of the strain of interest was inoculated into 5 mL LB medium. The cultures were grown overnight with shaking at 37°C. The following day, cultures were diluted to a starting turbidity at 600 nm of 0.05 (for β-galactosidase assays) or 0.005 (for luciferase assays) into 5 mL LB, and grown with shaking at 37°C to log phase (turbidity at 600 nm of 0.2, approximately 120 to 180 min, depending on media used). For induction, cells were generally pelleted by centrifugation (8K, 5 min) at room temperature, washed once in the same volume of prewarmed M9suppK medium at pH 7, then pelleted and resuspended in the same volume of prewarmed M9suppK at pH 7 or pH 5.5. Cells were generally grown for a further 30 min before assaying them for β-galactosidase or luciferase activity. In experiments testing the effect of endogenously produced indole, overnight cultures were diluted directly into the M9suppK medium at pH 7, and the pH of the induced culture was directly adjusted with a predetermined amount of 100 mM HCl to bring the pH to the correct final value once the cells had reached exponential phase.

### Promoter Probe Assays

β-Galactosidase assays and luciferase assays were done as described in Miller (1972) and Burton et al. (2010), respectively. All measurements were done at least in triplicate. Statistical analysis of differences between paired sets of data was done using unpaired *t*-tests; in all cases where a significant difference is referred to, *p*-values were less than 0.001.

### Measurement of Acid Resistance

Acid resistance assays were done as described (Johnson et al., 2014). All measurements were done at least in triplicate. Statistical analysis was done as described above.

### Measurement of IC50

The concentration of indole needed to inhibit low pH-mediated induction of *ydeP-lacZ* was determined by measuring the percentage inhibition of activity relative to activity in the presence of the same concentration of ethanol alone. Percentage inhibition was plotted vs. log_10_[indole concentration]. Curve fitting and determination of IC50 values was done using GraphPad Prism^1^ (GraphPad Software, La Jolla, CA, United States), using the option “log(inhibitor) vs. response – Variable slope (four parameters).”

### Autophosphorylation Assay

EvgS(557-1197)D1009A was expressed in BL21(DE3)/pETevgS(557-1197)D1009A grown in 2 × YT medium (1.6% tryptone, 1.0% yeast extract, 0.5% NaCl) and induced by 0.1 mM IPTG. Harvested cells were disrupted by sonication, and the protein was affinity purified by a Ni-NTA agarose column (the EvgS domain in this vector is expressed with a 6x His tail; it has been shown this has no effect on protein activity). Purified EvgS(557-1197)D1009A was autophosphorylated according to the conditions by Kinoshita-Kikuta et al. (2015). Briefly, 30 mM ATP was added to EvgS in the presence of 300 mM Tris-HCl (pH 8), 50 mM KCl, and 10 mM MgCl_2_ at 25°C, and the reaction terminated with the addition of 2 × sample buffer for SDS-PAGE. EvgS was separated with 10% SDS-PAGE, transferred to PVDF membrane (Immun-Blot, BioRad), and the membrane stained with Ponceau S to confirm the amount of protein transferred to the membrane. Phosphorylated EvgS was detected with anti-N3-pHis antibody (MerckMillipore), goat anti-rabbit IgG HRP (Abcam), and Immobilon Western Chemiluminescent HRP Substrate (MerckMillipore). For inhibition assays, indole was added to the reaction mixture at concentrations of 0, 50, 100, and 200 μM, and incubated at 25°C for 5 min prior to the addition of 30 mM ATP. Phosphorylation was continued for 60 min at 25°C, terminated with 2 × sample buffer, and analyzed as described above.

## Results

### Indole Inhibits Activation of Transcription From a Range of AR2 Promoters at Low pH

**FIGURE 1:**
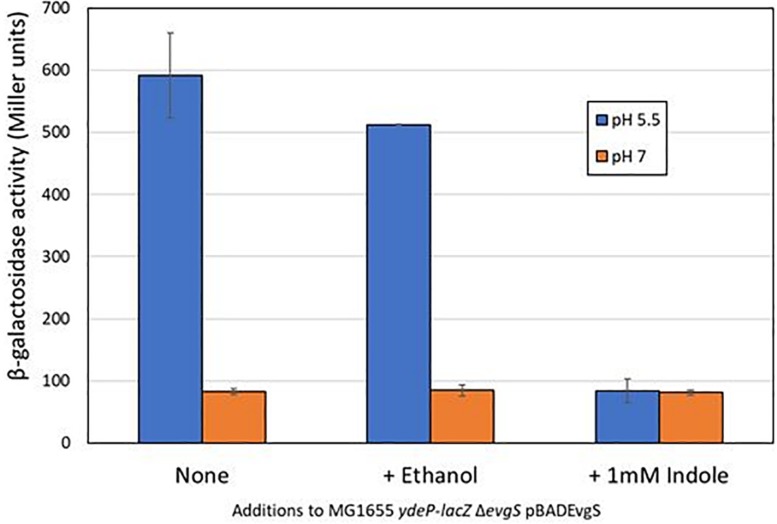
Indole inhibits the EvgS-dependent induction of *ydeP-lacZ* activity at pH 5.5. β-Galactosidase activity of *E. coli* MG1655 Δ*evgS ydeP-lacZ* pBADEvgS was measured following incubation at pH 5.5 or pH 7 with or without ethanol (final concentration 1%) or 1 mM indole dissolved in ethanol.

We initially used a chromosomal *ydeP-lacZ* operon fusion strain to investigate the effect of several different compounds (cadaverine, gamma-aminobutyric acid, glycine betaine, indole, ornithine, sarcosine, and spermine) on expression from the *ydeP* promoter, at a range of concentrations from 100 μM to 10 mM and at both pH 7 and pH 5.5. The *ydeP* promoter was chosen as it is induced very rapidly upon acidification, is directly regulated by the EvgA response regulator, and is not subject to complex regulation by other components of the AR2 network such as GadE, GadW, and GadX, or the stress induced sigma factor RpoS (Burton et al., 2010). The strain used for this assay lacked a chromosomal *evgS* gene. Functional EvgS [either wild-type or the constitutively “always on” protein EvgS-S600I which is active at pH 7 (Johnson et al., 2014)] was provided from a plasmid. Induction of the *ydeP-lacZ* fusion at pH 5.5 was dependent on the presence of EvgS, as expected. The presence of EvgS-S600I led to a much higher level of *lacZ* activity at pH 7, and clear induction of *ydeP* promoter activity at pH 5.5 was seen in the presence of both EvgS-S600I and wild-type EvgS (Supplementary Figure S1). Of the compounds tested, only indole showed any significant effect, completely blocking the induction of *ydeP-lacZ* at 1 mM. Ethanol alone (in which the indole was dissolved) was not responsible for this effect (Figure 1). To ensure that indole was not having an effect at low pH that might interfere with the use of *lacZ* as an effective promoter probe, we examined its effect on IPTG-induced β-galactosidase activity from the endogenous *lac* operon at both pH 7 and pH 5.5. The results (Supplementary Figure S2) showed that although the lower pH did depress activity somewhat, the presence of indole had no effect on this.

**FIGURE 2:**
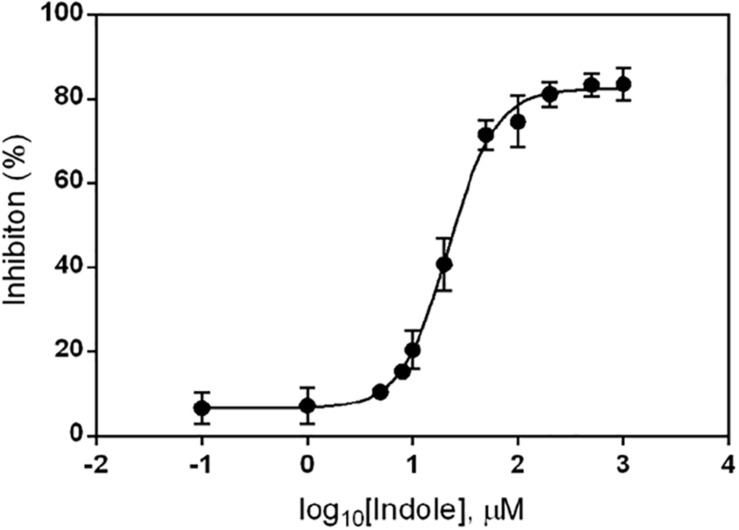
Inhibition of *ydeP-lacZ* reporter activity at different concentrations of indole. LacZ activity at pH 5.5 was measured in MG1655 Δ*evgS ydeP-lacZ* pBADEvgS over a range of indole concentrations and expressed as percentage inhibition of the value obtained in the absence of indole at pH 5.5. Curve fitting was done as described in section “Materials and Methods.”

To determine the concentration of indole needed to inhibit *ydeP* promoter induction by low pH, we examined its effect on *ydeP-lacZ* activity over a range of concentrations from 0.1 μM to 1 mM. The results, normalized as percentage inhibition of activity in the presence of ethanol alone, are shown in Figure 2. Fitting a binding curve to this gave an IC50 of 22 μM, and a Hill slope of 1.94, consistent with a co-operative effect.

**FIGURE 3:**
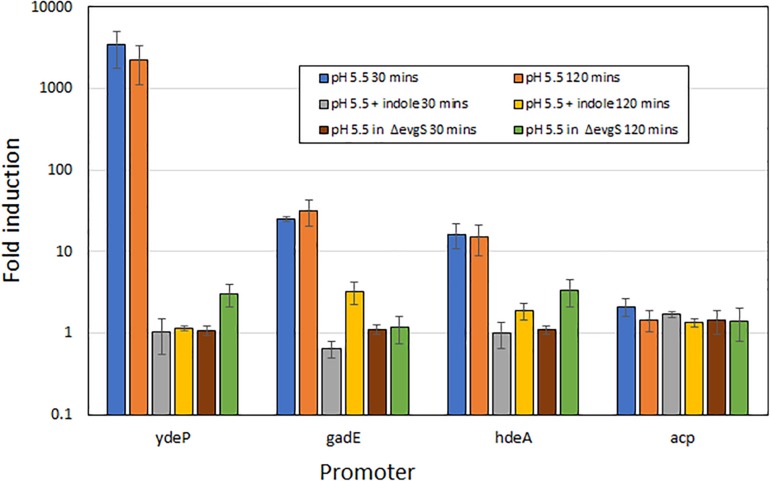
Induction of expression from AR2 promoters of *ydeP*, *hdeA*, and *gadE*, and the control promoter *acp*, at pH 5.5, measured using a luciferase reporter assay (Burton et al., 2010). Fold induction is expressed relative to the same promoter at pH 7, and values are shown for both 30 min and 120 min after acidification to pH 5.5 of exponential phase cultures. Indole was present at 1 mM.

To rule out the possibility that the effect of indole on the induction of AR2 gene expression was unique to the *ydeP* promoter, we investigated several other promoters of the AR2 system. To do this, we used a series of promoter probes that we had previously constructed where these promoters are fused to a luciferase operon on a low copy number plasmid (Burton et al., 2010). We tested three AR2 promoters with this system: *ydeP* (to validate the LacZ data), *gadE*, and *hdeA* (*hdeA* is directly regulated by GadE, and hence shows dependence on EvgAS as *gadE* expression is itself completely dependent on EvgAS; Burton et al., 2010). We also measured activity from the *acp* promoter, a control promoter that is not significantly induced at low pH. The results, shown in Figure 3, are expressed as log_10_ fold-induction at two different time points (relative to the same culture at pH 7) under the condition shown. These data show that the fold induction of the *ydeP* promoter was significantly higher than the others. The greater fold induction seen with the luciferase assay compared to the *lacZ* assay may reflect the greater dynamic range of this assay method. The results confirm that *ydeP*, *gadE*, and *hdeA* promoters all show induction of expression at pH 5.5, and that in each case, this induction is abolished in the presence of indole.

### Exogenously Produced Indole Inhibits *ydeP* Promoter Activity and Blocks AR2-Mediated Acid Resistance

**FIGURE 4:**
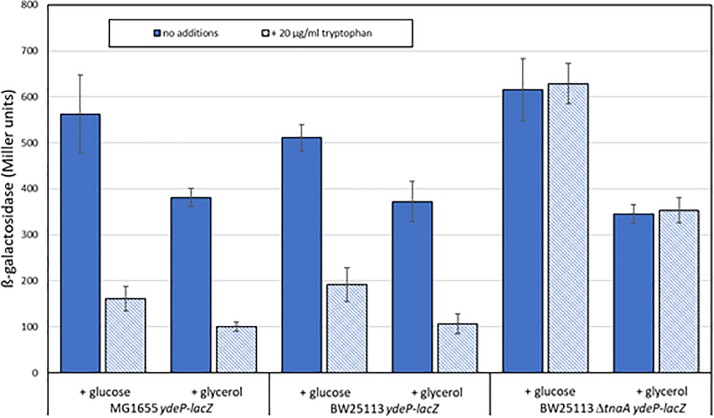
Tryptophan inhibits the acid induction of EvgS activity in a *tnaA*-dependent fashion. The activity of the *ydeP*-*lacZ* fusion was determined in MG1655, BW25113, and BW25113 Δ*tnaA*, all measurements following induction at pH 5.5, in the presence or absence of 100 μM tryptophan. Carbon sources were glucose or glycerol as shown.

*Escherichia coli* produces indole solely from tryptophan via a reaction catalyzed by tryptophanase, the product of the *tnaA* gene. The amount of indole produced is directly proportional to the level of tryptophan in the growth medium (Lee and Lee, 2010; Li and Young, 2013). Levels of indole vary during the course of growth with a high-level pulse of cell-associated indole being seen as strains transition to stationary phase (Gaimster et al., 2014). Our standard growth and induction medium lacks tryptophan, as it is broken down by the acid hydrolysis that produces casamino acids, and is presumed not to contain any indole. We therefore reasoned that the addition of tryptophan to the growth medium might block AR2 induction in a *tnaA*-dependent manner, as wild type cells would metabolize it to indole, and that this effect would be blocked in a *tnaA* mutant. We tested this using the *ydeP-lacZ* reporter in MG1655, and in other strains constructed by transducing this fusion into either BW25113 or into the Keio library knockout derivative BW25113 *tnaA*:*kan*^R^. Assays were done as before, except that cultures were grown in a M9suppK pH 7 medium after overnight dilution from LB. Cultures were acidified in early logarithmic phase by adding a calibrated amount of hydrochloric acid directly to the medium, rather than by spinning and re-suspending, to avoid discarding any indole produced during bacterial growth up to that point. As glucose has been reported to repress tryptophanase activity (Freundlich and Lichstein, 1960), we did experiments with either glucose or glycerol as a carbon source. The results (Figure 4) confirm both of the above predictions, and in addition show that the effect of choice of carbon source on the induction of *ydeP-lacZ* is small, although activities are generally lower when cells are grown with glycerol as a sole carbon source. The lack of glucose repression may reflect the fact that repression of TnaA activity at the concentration of glucose used here is only around 50% (Freundlich and Lichstein, 1960), so the residual level of activity may be sufficient to produce enough indole to cause the inhibitory effect seen here.

**FIGURE 5:**
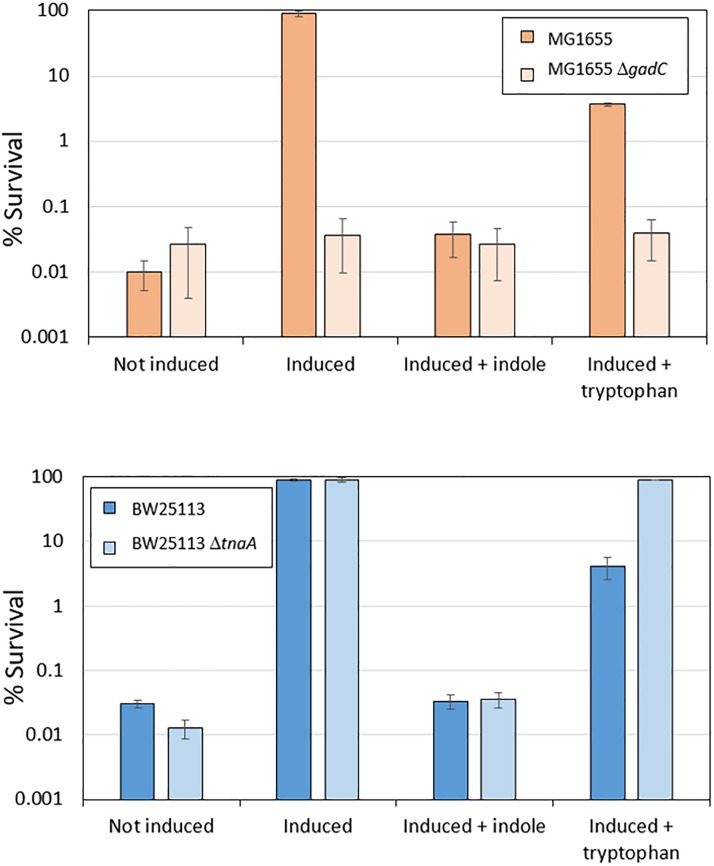
Induced acid resistance is suppressed by indole. Percentage survival values at pH 2.5 of different *E. coli* strains (as shown) were determined as described in section “Materials and Methods,” with or without induction at pH 5.5 (labeled “induced” or “not induced” respectively) in the presence or absence of 1 mM indole or 100 μM tryptophan.

When *E. coli* cells are exposed to mild acid stress at pH 5.5, activation of EvgS induces the AR2 system, which then confers resistance against subsequent exposure to an extreme acid stress at pH 2.5. Given the above finding that AR2 induction can be inhibited by indole produced by growing cells, we predicted that the addition of indole or tryptophan would also block induced acid resistance, but that the effect of tryptophan would not be seen in a *tnaA* mutant. To investigate this, we first confirmed that the addition of indole to a culture of MG1655 cells grown in a M9suppK medium reduced the ability of these cells to survive exposure to pH 2.5 to the same level as a *gadC* deletion mutant, which prevents the AR2 system from operating (Figure 5). We then showed that loss of induced acid resistance was seen when tryptophan was added to this growth medium, although the effect was not as pronounced, possibly because at the stage when the cells were exposed to the mild acid shock of pH 5.5, not all the tryptophan had been converted to indole and so the inhibitory effect was reduced. We then investigated the effect of a *tnaA* mutant derivative of the strain BW25113. High acid resistance was induced in BW25113 at pH 5.5, and this was completely blocked by the addition of indole (irrespective of the presence or absence of *tnaA*) (Figure 5). Addition of tryptophan led to a partial suppression of induced acid resistance in BW25113, consistent with the hypothesis proposed above that only partial conversion of tryptophan to indole had occurred at the time when the assay was done. Tryptophan completely failed to suppress resistance in a *tnaA* mutant, as predicted.

A persistent and unexplained finding in the literature is that AR2 is not induced when cells are incubated in LB at pH 5.5. This could be because the action of tryptophanase on the tryptophan present in LB results in indole levels that reach between 340 μM and 1 mM at a stationary phase, and between 0.1–0.2 μM after only 2 h of growth (Wang et al., 2001; Li and Young, 2013; Gaimster et al., 2014). If this is the sole cause of inhibition of AR2, a *tnaA* mutant would not show this phenotype. We tested this by measuring *ydeP-lacZ* activity in both BW25113 and BW25113 Δ*tnaA* after growth in LB. No induction was seen, even in the *tnaA* mutant, so we conclude that conversion of endogenous tryptophan to indole is not solely responsible for the failure of AR2 to become activated at pH 5.5 in LB (Supplementary Figure S3).

### EvgS Is the Likely Target of Indole Inhibition

An attractive hypothesis for the effect of indole on AR2 induction is that it is inhibiting one of the activators of AR2 gene expression. There are only two known regulators in common between *gadE* and *ydeP*: the response regulators EvgA and RcsB. Loss of either of these leads to loss of acid-mediated induction of transcription from both of these promoters in exponential phase (Burton et al., 2010; Johnson et al., 2014); thus, indole could in principle be inhibiting the activity of either, or both, of these regulators.

We have previously shown that induction of AR2 promoter activities at pH 5.5 is lost in an *rcsB* knock-out. Moreover, these promoter activities are not restored in the presence of a constitutively active EvgS protein that is active even at pH 7 (Johnson et al., 2014). We therefore examined the effect of indole on the expression of *ydeP-lacZ* in the presence of such a protein, EvgS S600I. The results showed that the inhibitory effect of indole on the induction of *ydeP* by low pH was completely suppressed when EvgS S600I was expressed (Supplementary Figure S4), ruling out the possibility that indole is acting on RcsB. This therefore supports the alternative hypothesis that indole directly or indirectly inhibits some aspect of EvgS activation, though other more complex hypotheses, considered in the Discussion, are possible.

**FIGURE 6:**
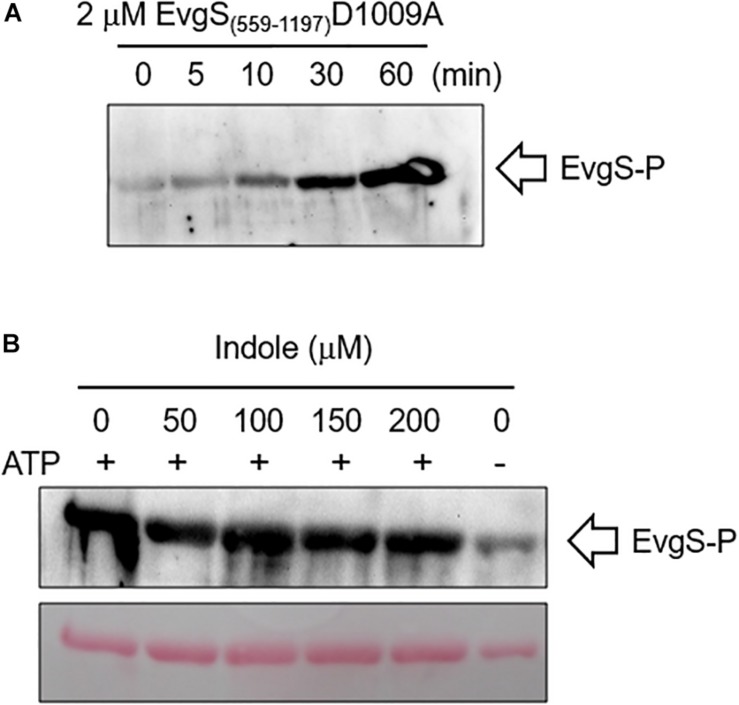
Indole does not inhibit *in vitro* autophosphorylation of EvgS. **(A)** 2 μM of EvgS(557-1197)-D1009A was autophosphorylated with 30 mM ATP in EvgS buffer (300 mM Tris-HCl (pH8), 50 mM KCl, 10 mM MgCl_2_) at 25°C. EvgS was separated on a 10% SDS-PAGE gel, and phosphorylation was analyzed by immunoblotting with anti-N3-pHis antibody (first antibody) and anti-rabbit IgG-HRP (second antibody). Signals were detected with Immobilon Western (Millipore). Figures above the gel show minutes of incubation. **(B)** 2 μM of EvgS(557-1197) D1009A was autophosphorylated as in A for 60 min in the absence and presence of different concentrations of indole as indicated. EvgS was separated by SDS-PAGE, and phosphorylation analyzed as in **(A)**. Upper panel: immunoblotting. Lower panel: Ponceau S stain (loading control).

As a further check of this result, we measured the effect of indole on autophosphorylation of EvgS *in vitro* using a purified cytoplasmic domain of EvgS (amino acids 557–1197). A form of the protein with the substitution D1009A was used for this assay, as this stabilizes the phosphorylated form by blocking the internal phosphorelay. It has been shown that autophosphorylation of this protein *in vitro* occurs only at H721 (Kinoshita-Kikuta et al., 2015). The purified protein showed autophosphorylation *in vitro* as expected, and this was not affected by any of the indole concentrations tested (Figure 6). Thus, if indole is indeed inhibiting EvgS activation, both biochemical and genetic data show it must be acting upstream of the phosphorylation step.

### Collapse of the Proton Gradient Does Not Prevent AR2 Promoter Activation by Low pH

**FIGURE 7:**
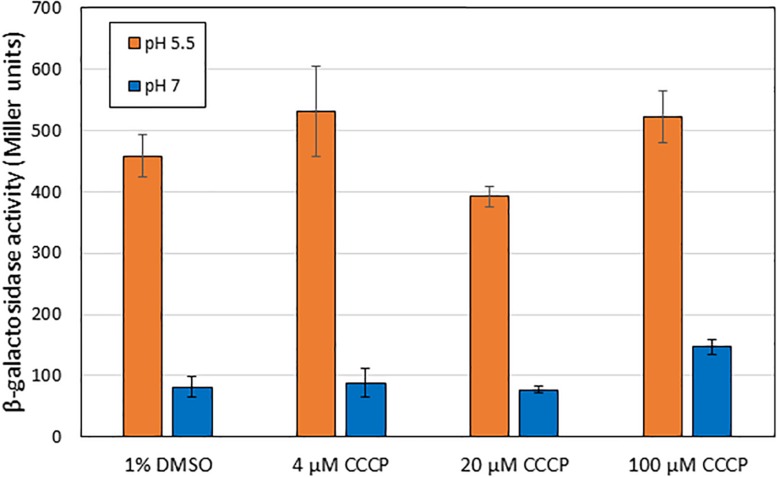
Addition of CCCP does not block activation of EvgS at pH 5.5. β-Galactosidase activity was measured in MG1655 Δ*evgS ydeP-lacZ* pBADEvgS after induction at pH 5.5 or pH 7 in the presence of CCCP dissolved in DMSO, or DMSO alone, at the indicated concentrations.

One hypothesis for the induction mechanism of the AR2 system is that it is activated by the difference between periplasmic and cytoplasmic pH, rather than by a low periplasmic pH *per se*. This could come about if, for example, EvgS acted as a sensor of the proton motive force. Indole has been reported to act as an ionophore and to cause progressive loss of *E. coli* membrane polarization at sufficiently high concentrations (>2 mM, Chimerel et al., 2012; Krasnopeeva et al., 2019). Although the concentrations at which indole caused inhibition of AR2 activity are well below this, we wanted to test whether the collapse of the proton gradient mediated by a different compound caused the inhibition of induction of AR2 activity. For this purpose, we used the ionophore CCCP, which shuttles protons across membranes and hence collapses transmembrane pH gradients (Kasianowicz et al., 1984; Kinoshita et al., 1984). Based on values reported in other studies, we tested the effects of CCCP at three concentrations (4 μM, 20 μM, and 100 μM). As the results in Figure 7 show, even at quite high concentrations, CCCP had little effect on the induction of the *ydeP* promoter at pH 5.5. We measured the logarithmic growth rates of *E. coli* under different concentrations of indole and CCCP used in these experiments and showed that CCCP completely blocked *E. coli* growth at sufficiently high concentrations, whereas indole did not (Supplementary Table S2). This is consistent with published data showing that indole only blocks *E. coli* growth at concentrations greater than 3 mM (Chant and Summers, 2007), whereas inhibitory concentrations of CCCP have been reported in the range 10–50 μM, depending on strain and media conditions (Kinoshita et al., 1984; Ghoul et al., 1989; Chant and Summers, 2007). The inhibition of growth presumably is because the cells are not able to generate sufficient ATP. Conversely, indole had no measurable effects on growth rate even at concentrations that completely inhibited induction of the *ydeP* promoter. We conclude that the effect of indole on the AR2 system cannot be explained in terms of its effect as an ionophore, and, moreover, that the AR2 system is not being activated under the conditions used here as a consequence of the cell detecting the change in the proton gradient that occurs when the periplasm is acidified.

## Discussion

Two component systems enable bacteria to integrate information about changes in their external environments and to rapidly respond to these (Mascher et al., 2006; Gao and Stock, 2009; Krell et al., 2010; Zschiedrich et al., 2016; Jacob-Dubuisson et al., 2018). Despite extensive research on their structure and function, the mechanisms whereby the sensor kinases detect a signal are well understood in only a small number of cases. They appear to be a mixture of direct (binding of a specific ligand) and indirect (sensing some attribute of the cell). Some involve altering protein conformation in response to cellular conditions such as its redox or osmotic state, while others involve binding of a specific ligand or ligands. Given the prevalence of activation and repression loops in regulatory circuits, it would not be surprising to discover sensor kinases that are regulated by multiple signals that either activate or repress their activity. Repression mechanisms could vary from direct or indirect blocking of access to an activating ligand, through inhibition of a conformational change required as some part of the signal transduction mechanism, to a shift in the equilibrium from the kinase toward the phosphatase form, that catalyze either removal of the phosphates from the response regulator or the kinase itself. Known examples of such mechanisms include the BvgS kinase in *Bordetella pertussis*, a close homolog of EvgS. This kinase is inhibited by nicotinate, that binds to one of the periplasmic Venus fly-trap domains and, in doing so, shifts BvgS to the phosphatase state (Dupré et al., 2015b). In *E. coli*, the response regulator ArcB (which also shares some homology with EvgS) is shifted between inactive and active states by the relative oxidation states of the quinone pool (Malpica et al., 2004; Bekker et al., 2010). However, the number of known specific inhibitors of sensor kinase activity is small. Identification of compounds that inhibit the activity of two component systems has been mooted as a potential path to find new antibiotics (see for example, Desnottes, 1996; Matsushita and Janda, 2002; Watanabe et al., 2008; Gotoh et al., 2010; Bem et al., 2015). Indeed, one such compound, waldiomycin, which was identified from a screen for kinase inhibitors, has been proposed as a potential treatment of Gram-positive bacterial infections (Igarashi et al., 2013; Fakhruzzaman et al., 2015; Eguchi et al., 2017). Other examples of identified TCS inhibitors are given in Bem et al. (2015).

We have shown here that indole acts to inhibit the pH-mediated activation of several genes in AR2, which is part of the EvgS regulon, and that this effect occurs upstream of the autophosphorylation step of EvgS. The simplest explanation that is consistent with our data is that indole is acting directly or indirectly on EvgS itself. Indole could, for example, bind directly to EvgS and inhibit its ability to detect its inducing signal, or its ability to transduce information about that detection. Alternatively, it could alter the pH range that EvgS detects. The low concentration at which the inhibitory effect is seen makes direct binding of indole to EvgS an attractive model. But indole could also alter a property of the cell that is responsible for AR2 activation. We have ruled out the possibility that this inhibition is due to indole perturbing the transmembrane proton gradient, by showing that it inhibits induction of AR2 at a much lower concentration than that required to cause such a perturbation, and by showing that an agent that does perturb the gradient (CCCP) does not inhibit AR2 induction. The *in vitro* kinase activity of EvgS and BvgS are both strongly inhibited by oxidized ubiquinone (by direct reduction in kinase activity, rather than activation of phosphatase activity; Bock and Gross, 2002), and it is conceivable that indole could perturb the balance of oxidized and reduced quinones in the quinone pool and, thus, affect EvgS activity, but again the demonstration that the impact of indole is likely to be upstream of the kinase activity makes this explanation, in our view, unlikely. More complex hypotheses are also possible; for example, indole could inhibit an unknown regulator that activates AR2. The fact that the constitutively active EvgS protein EvgS S600I can suppress the inhibition caused by indole also makes such hypotheses less likely. Distinguishing these different possibilities will require further genetic and biochemical studies, and these are currently underway.

Several previous studies have linked indole with aspects of acid resistance in *E. coli*. A micro-array analysis of gene expression in indole-treated biofilms revealed the repression of several genes of the AR2 system (including *gadE* and *hdeA*, also tested in this study), and the protein YmgB was shown to be responsible for mediating this effect (Lee et al., 2007a, b). These authors also demonstrated that acid resistance was reduced by indole treatment. Their method of determining acid resistance did not involve pre-incubation in an inducing medium within the pH range that EvgS responds to, and strains in this study were grown in LB, so it is unlikely that they were studying the same phenomenon as we report here. In a separate study, treatment with 1 mM or 2 mM indole caused an increase in *E. coli* survival at pH 3.5 (Hirakawa et al., 2010). Although this differs from our findings, the study was done using LB-grown *E. coli* without pre-induction of the acid resistance response at pH 5.5, and was done in the strain MC4100 which carries a substitution in EvgS that renders it unresponsive to low pH (Eguchi and Utsumi, 2014), so the studies are not directly comparable. Indole has also previously been shown to induce a range of drug exporter genes, as well as the central AR2 regulator GadE (Hirakawa et al., 2005), and the role of EvgS was explicitly ruled out in this study (which was also done using  MC4100 grown in LB). We conclude from comparing these studies with the present work that indole can affect acid resistance in *E. coli* in multiple ways and via multiple routes. Ongoing studies in this field need to be carefully designed with strain and media influences in mind.

Indole has long been known to be a metabolic product of *E. coli*; indeed, a positive test for indole production is often a feature of rapid clinical diagnostic tests for *E. coli* infections (Edberg and Trepeta, 1983). In recent years, the importance of indole’s role as a signaling molecule has become more obvious. Among other things, indole has been shown to play a role in signaling associated with a range of important bacterial phenotypes including biofilm formation, motility, cell adherence, cell division, plasmid stability, drug resistance, virulence, persister cell formation, and others, in both laboratory strains and pathogenic strains of *E. coli* (Bansal et al., 2007; Lee et al., 2009; Lee and Lee, 2010). Indole is produced at low levels during bacterial growth, but a surge of production that can lead to very high intracellular levels (up to 60 mM) has been associated with the transition from exponential to stationary phase (Gaimster et al., 2014; Gaimster and Summers, 2015). Indole can diffuse across bacterial membranes but may also partition into them, which could lead to elevated levels of indole in the vicinity of *trans*-membrane proteins, even if the extracellular levels are relatively low (Piñero-Fernandez et al., 2011). Intriguingly, it has also been shown recently that indole has a role in the regulation of the internal pH of *E. coli*, with the stationary phase pulse of indole signaling setting the internal pH to 7.2, as opposed to 7.8 in the absence of indole (Zarkan et al., 2019). The concentration of indole and how it varies in the human gut is unknown, but high levels (250–1000 μM) are typically found in stool samples (Karlin et al., 1985). This is because many gut bacteria are indole producers, and indole and tryptophan are both common components of many foods. Given the complexity of the interactions between indole and experimental strains of *E. coli* grown under laboratory conditions, a full understanding of the implications of the interaction of indole with commensal and pathogenic *E. coli*, and with other members of the gut microbiota, is still remote. Indole may be an additional cue that enables bacteria to modulate gene expression in order to optimize their survival in the gut. Indeed, a recent study has shown a mechanism whereby indole levels in the mouse gut can directly modulate the expression of pathogenesis genes in enterohemorrhagic *E. coli*, via the CpxA two component system sensor kinase (Kumar and Sperandio, 2019). The GAD genes, regulated in part by EvgS, have also been implicated in some aspects of pathogenicity of *E. coli* in a number of studies (e.g., Tree et al., 2011; Branchu et al., 2014), so understanding the novel effects of indole reported in this paper may enhance our understanding of the complex interactions between *E. coli* and the gut environment that lead to colonization and, in some cases, infection. It is possible, for example, that indole could act as a positional cue for *E. coli*, enabling it to regulate gene expression to be appropriate to its location in the gut, and that EvgS may in part be responsible for the co-ordination of this process.

## Data Availability Statement

The datasets generated for this study are available on request to the corresponding author.

## Author Contributions

NB, MK, AA, and MB did all the strain constructions and experiments involving measurement of lacZ and luciferase activities. KS did the experiments on autophosphorylation. YE and PL conceived of and supervised the work and wrote the manuscript.

## Conflict of Interest

The authors declare that the research was conducted in the absence of any commercial or financial relationships that could be construed as a potential conflict of interest.

## Footnotes

1 www.graphpad.com

## Supplementary Material

FIGURE S1 Expression of the *ydeP-lacZ* reporter is induced at pH 5.5 in an EvgS-dependent fashion. MG1655 Δ*evgS ydeP-lacZ* containing the plasmids shown was grown to log phase, then incubated at pH 5.5 or pH 7 for 30 min, and levels of β-galactosidase were determined. EvgS-S600I encodes a constitutively active version of EvgS which is active at pH 7 [14].

FIGURE S2 Indole has no effect on the activity of endogenous β-galactosidase at pH 7 or pH 5.5. The endogenous *lac* operon in MG1655 was induced by incubation for 30 min with 1 mM IPTG in the presence or absence of 1 mM indole, and the resulting levels of β-galactosidase were determined.

FIGURE S3 EvgS activity is not induced in LB irrespective of the presence or absence of *tnaA*. The activity of the *ydeP-lacZ* fusion in BW25113 was determined in the presence or absence of the tnaA gene after growth and induction in LB as described in section “Materials and Methods.”

FIGURE S4 Indole does not block the constitutive activity of EvgS S600I. β-galactosidase activity was measured in exponential phase cultures of MG1655 Δ*evgS ydeP-lacZ* pBADEvgS-S600I at pH 7, grown in the presence or absence of 1 mM indole.

TABLE S1 Strains and plasmids used in this study.

TABLE S2 Comparison of growth rates under conditions used for promoter probe assays in the presence of indole or CCCP. Assays were done at least in triplicate; growth was at 37°C in M9suppK medium.
