# Supplementary figures and images for: The Signaling Molecule Indole Inhibits Induction of the AR2 Acid Resistance System in Escherichia coli
Source: Front Microbiol. 2020 Apr 15;11:474. doi: 10.3389/fmicb.2020.00474 (PMC7174508; doi:10.3389/fmicb.2020.00474)

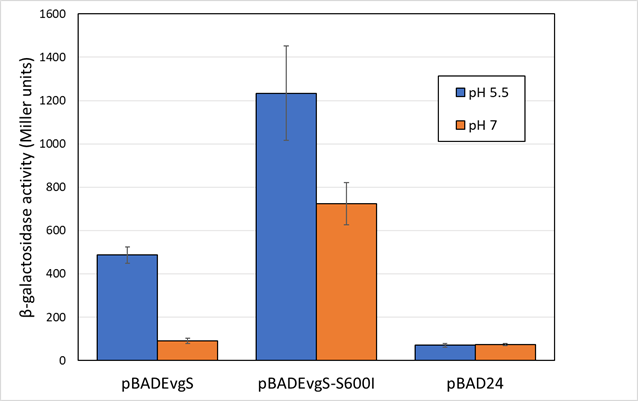

Supplement: FIGURE S1 — Expression of the ydeP-lacZ reporter is induced at pH 5.5 in an EvgS-dependent fashion. MG1655 ΔevgS ydeP-lacZ containing the plasmids shown was grown to log phase, then incubated at pH 5.5 or pH 7 for 30 min, and levels of β-galactosidase were determined. EvgS-S600I encodes a constitutively active version of EvgS which is active at pH 7 [14]. [file Image_1.TIF]

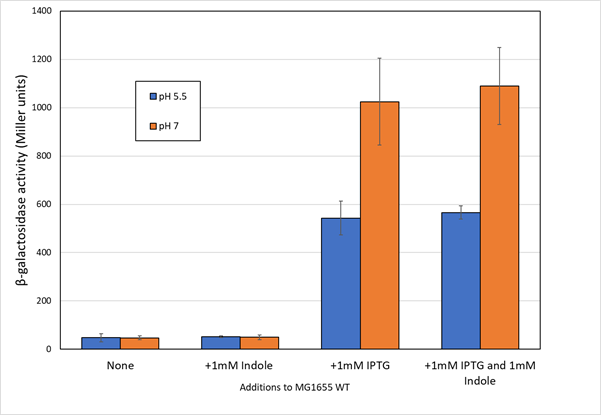

Supplement: FIGURE S2 — Indole has no effect on the activity of endogenous β-galactosidase at pH 7 or pH 5.5. The endogenous lac operon in MG1655 was induced by incubation for 30 min with 1 mM IPTG in the presence or absence of 1 mM indole, and the resulting levels of β-galactosidase were determined. [file Image_2.TIF]

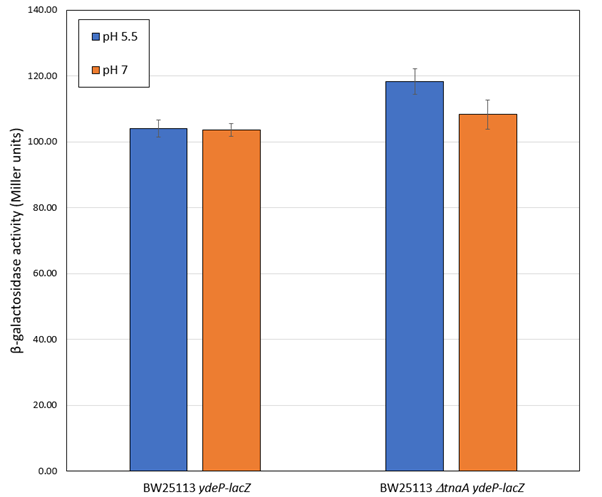

Supplement: FIGURE S3 — EvgS activity is not induced in LB irrespective of the presence or absence of tnaA. The activity of the ydeP-lacZ fusion in BW25113 was determined in the presence or absence of the tnaA gene after growth and induction in LB as described in section “Materials and Methods.” [file Image_3.TIF]

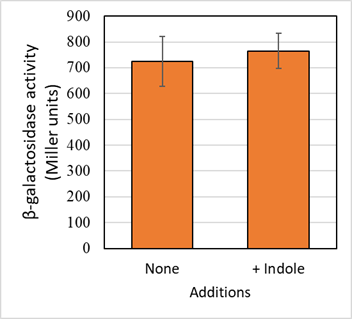

Supplement: FIGURE S4 — Indole does not block the constitutive activity of EvgS S600I. β-galactosidase activity was measured in exponential phase cultures of MG1655 ΔevgS ydeP-lacZ pBADEvgS-S600I at pH 7, grown in the presence or absence of 1 mM indole. [file Image_4.TIF]
