# Supplementary material for: The Signaling Molecule Indole Inhibits Induction of the AR2 Acid Resistance System in Escherichia coli
Source: Front Microbiol. 2020 Apr 15;11:474. doi: 10.3389/fmicb.2020.00474 (PMC7174508; doi:10.3389/fmicb.2020.00474)
Supplement: TABLE S1 — Strains and plasmids used in this study. [file Table_1.pdf]

Table S1: Strains and plasmids used in this study

**Strains**	**Description**	**Reference**
MG1655	*E. coli* K12 F^- λ^- *rph*	
MG1655 ~~p~~*ydeP-lacZ*	Chromosomal *ydeP-lacZ* operon fusion with linked *kan*^{R} marker	Eguchi and Utsumi, 2014
MG1655 Δ*evgS::cat*	Chromosomal *evgS* gene inactivation by insertion of chloramphenicol resistance gene	Eguchi and Utsumi, 2014
MG1655 Δ*evgS::cat* p*ydeP-lacZ*	Strain carrying *ydeP-lacZ* operon fusion and inactivation of *evgS* on chromosome	Eguchi and Utsumi, 2014
MG1655 Δ*gadC*	Gene for GadC antiporter (required for AR2) deleted by P1 transduction from Keio library	Burton *et al.*, 2010
BW25113	F^- Δ(*araD-araB*) *lacZ* λ^- *rph* Δ(*rhaD-rhaB*) *hsdR*	Datsenko and Wanner, 2000
BW25113 p*ydeP-lacZ kan*^{R}	Constructed from BW25113 by P1 transduction	This work
BW25113 Δ*tnaA::kan*^{R}	From Keio collection	Baba *et al.*, 2006
BL21(DE3)	Strain for protein expression	Novagen
**Plasmids**	**Description**	**Reference**
pBAD24	Cloning vector for expression from pBAD promoter, encodes ampicillin resistance	Guzman *et al.*, 1995
pBAD24-EvgS	His-tagged EvgS expressed from pBAD promoter in pBAD24	Eguchi and Utsumi, 2014

pBAD24-EvgS.S600I	EvgS-S600I expressed from pBAD promoter in pBAD24	Johnson *et al*. 2014
pLux-*xxx*	Family of plasmids expressing bacterial luciferase under the control of promoter for gene *xxx*	Burton *et al*., 2010
pETevgS(557-1197)D1009A	Cytoplasmic region of EvgS with D1009A mutation expressed from T7 promoter in pET21a(+)	Kinoshita-Kikuta *et al*., 2015
