# Supplementary material for: The Signaling Molecule Indole Inhibits Induction of the AR2 Acid Resistance System in Escherichia coli
Source: Front Microbiol. 2020 Apr 15;11:474. doi: 10.3389/fmicb.2020.00474 (PMC7174508; doi:10.3389/fmicb.2020.00474)
Supplement: TABLE S2 — Comparison of growth rates under conditions used for promoter probe assays in the presence of indole or CCCP. Assays were done at least in triplicate; growth was at 37°C in M9suppK medium. [file Table_2.pdf]

Table S2. Comparison of growth rates under conditions used for promoter probe assays in the presence of indole or CCCP. Assays were done at least in triplicate.

culture conditions	mean growth rate (/hr)	St dev
pH 7	0.82	0.04
pH 7 + DMSO	0.85	0.04
pH 7 + 4 μM CCCP	0.85	0.07
pH 7 + 20 μM CCCP	<0.06	<0.006
pH 7 + 100 μM CCCP	<0.06	<0.006
pH 7 + Ethanol	0.82	0.06
pH 5.5 + ethanol	0.82	0.01
pH 5.5 + 10 μM indole	0.79	0.07
pH 5.5 + 100 μM indole	0.85	0.03
pH 5.5 + 1000 μM indole	0.84	0.02
